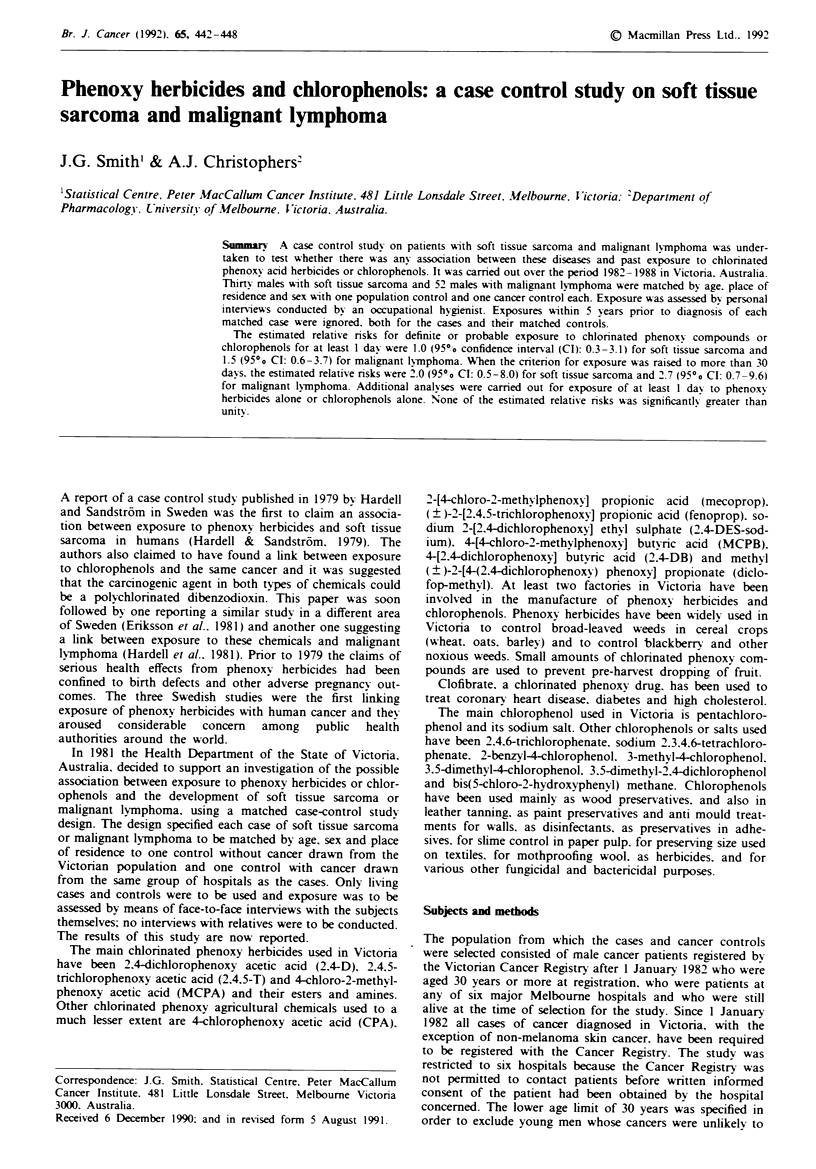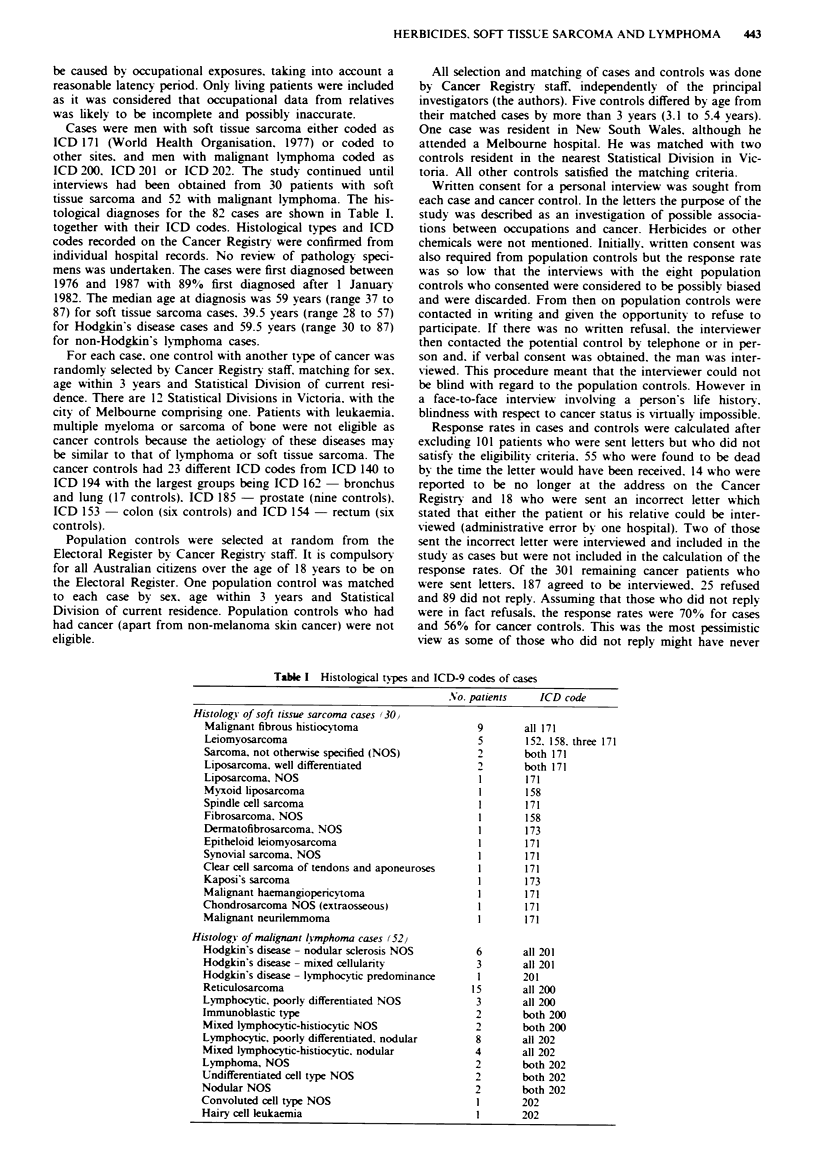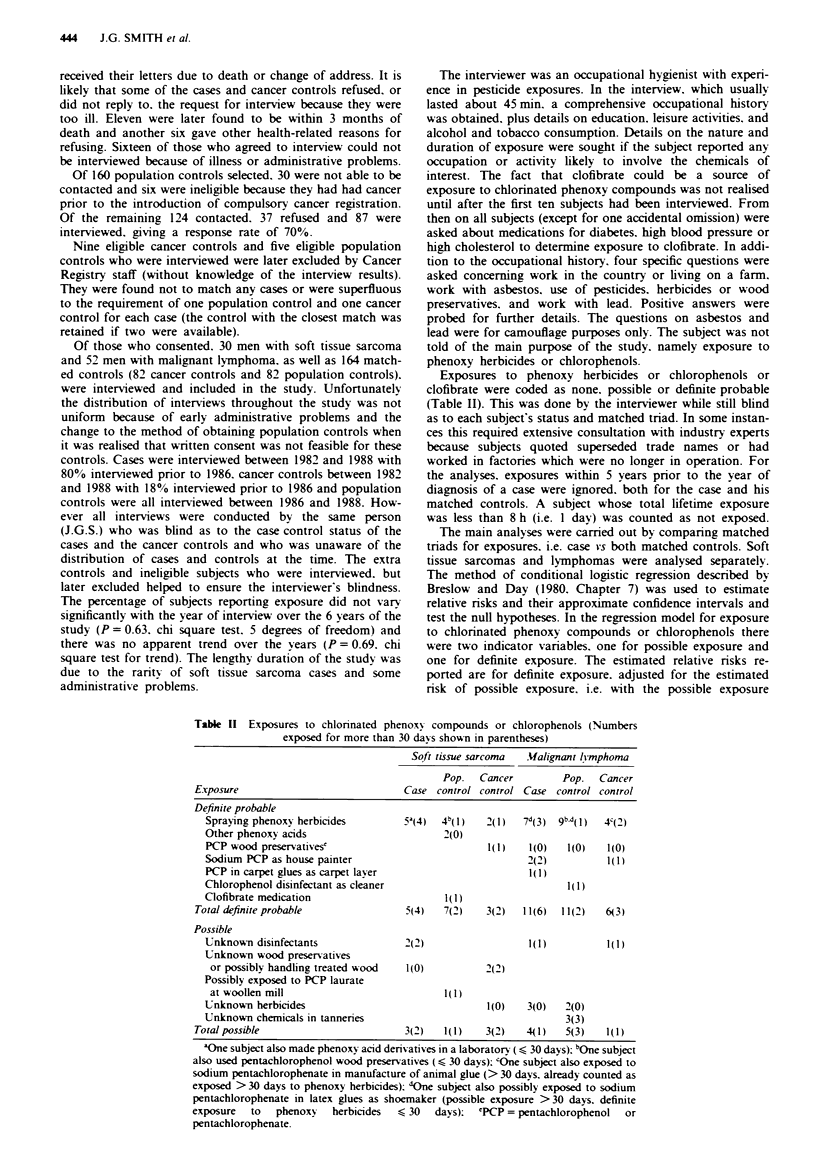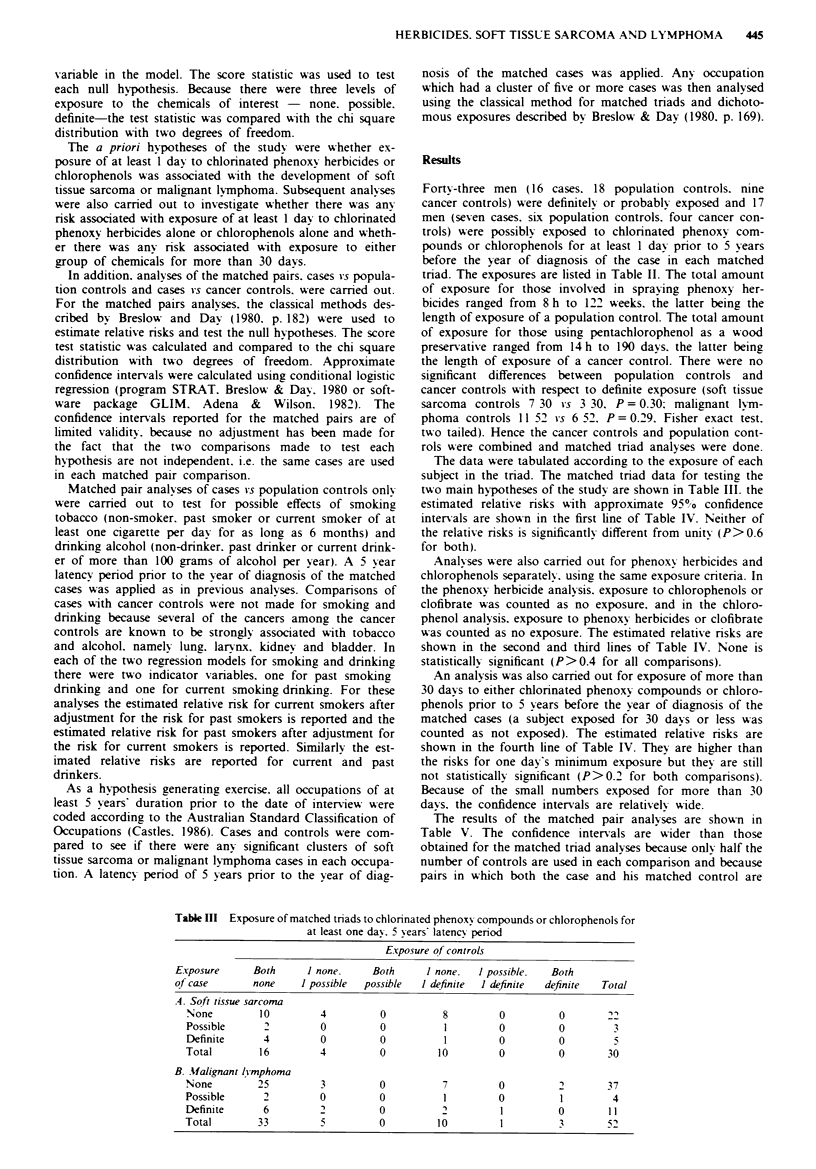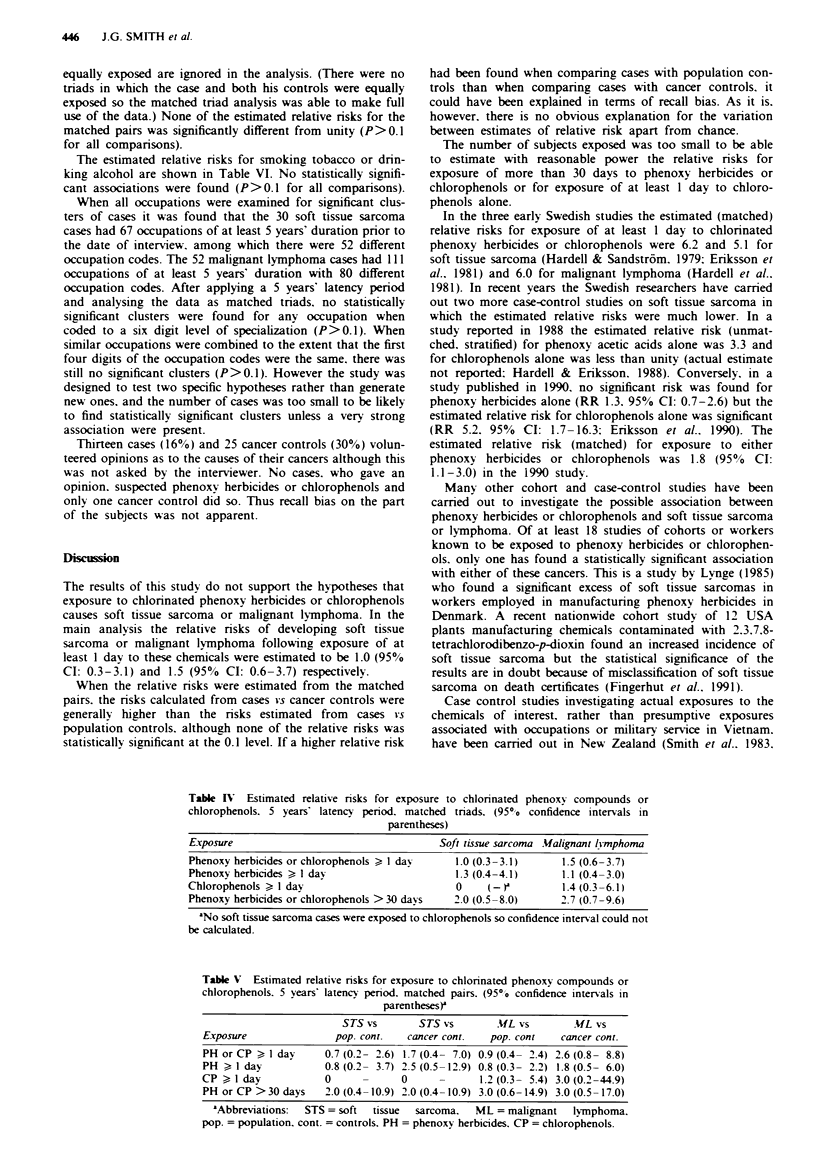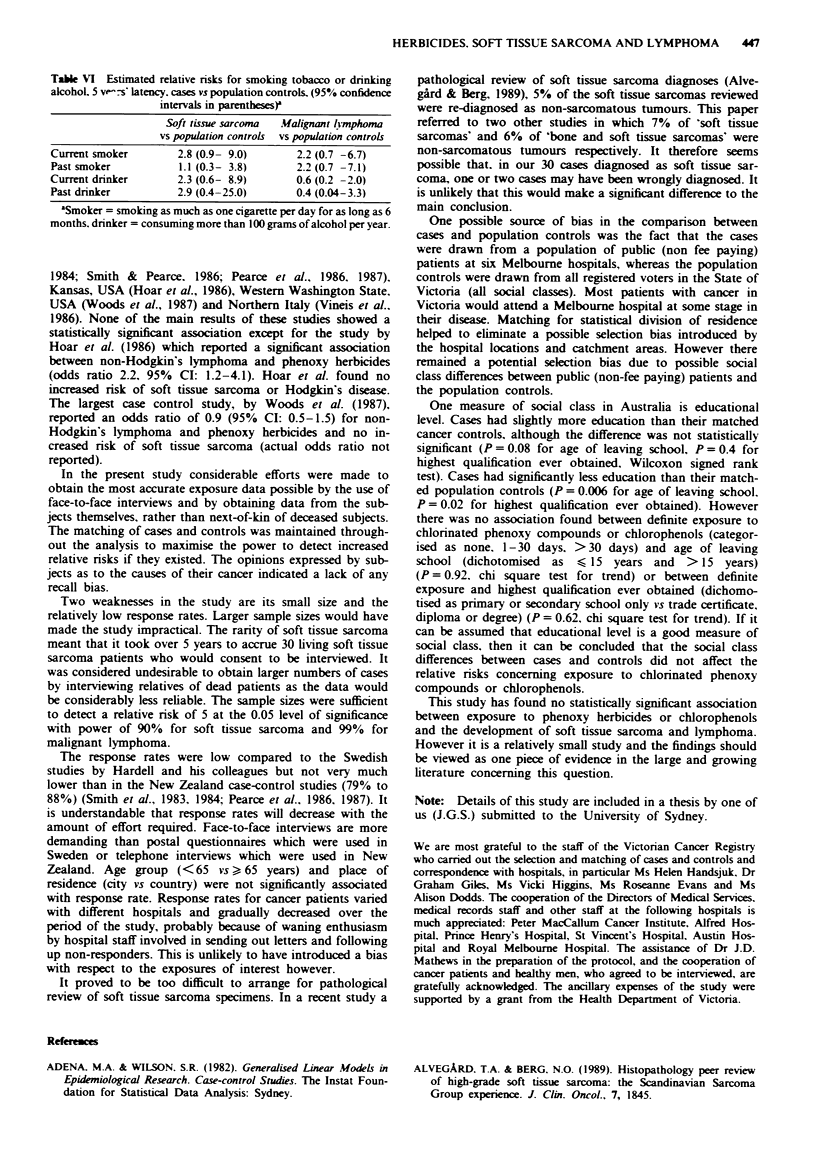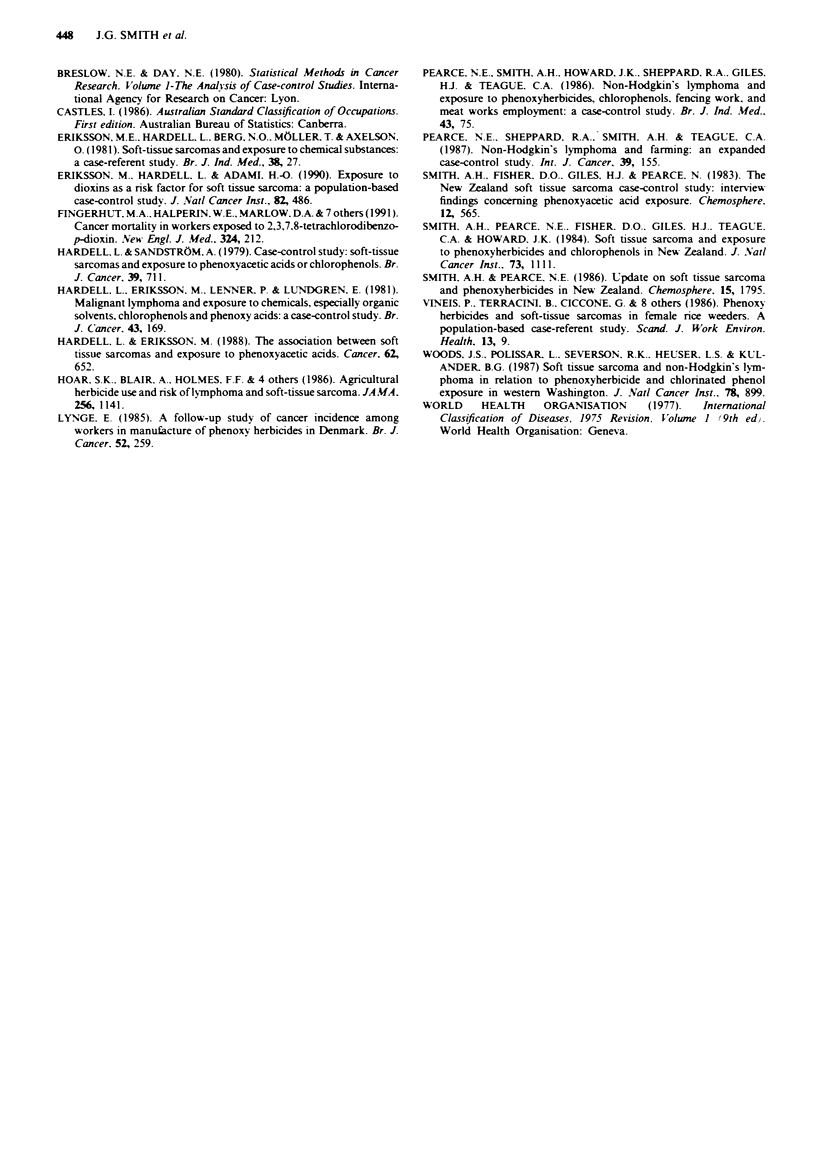# Phenoxy herbicides and chlorophenols: a case control study on soft tissue sarcoma and malignant lymphoma.

**DOI:** 10.1038/bjc.1992.90

**Published:** 1992-03

**Authors:** J. G. Smith, A. J. Christophers

**Affiliations:** Statistical Centre, Peter MacCallum Cancer Institute, Melbourne, Victoria, Australia.

## Abstract

A case control study on patients with soft tissue sarcoma and malignant lymphoma was undertaken to test whether there was any association between these diseases and past exposure to chlorinated phenoxy acid herbicides or chlorophenols. It was carried out over the period 1982-1988 in Victoria, Australia. Thirty males with soft tissue sarcoma and 52 males with malignant lymphoma were matched by age, place of residence and sex with one population control and one cancer control each. Exposure was assessed by personal interviews conducted by an occupational hygienist. Exposures within 5 years prior to diagnosis of each matched case were ignored, both for the cases and their matched controls. The estimated relative risks for definite or probable exposure to chlorinated phenoxy compounds or chlorophenols for at least 1 day were 1.0 (95% confidence interval (CI): 0.3-3.1) for soft tissue sarcoma and 1.5 (95% CI: 0.6-3.7) for malignant lymphoma. When the criterion for exposure was raised to more than 30 days, the estimated relative risks were 2.0 (95% CI: 0.5-8.0) for soft tissue sarcoma and 2.7 (95% CI: 0.7-9.6) for malignant lymphoma. Additional analyses were carried out for exposure of at least 1 day to phenoxy herbicides alone or chlorophenols alone. None of the estimated relative risks was significantly greater than unity.


Br. J. Cancer (1992), 65, 442-448 © Macmillan Press Ltd., 1992

### Phenoxy herbicides and chlorophenols: a case control study on soft tissue sarcoma and malignant lymphoma

J.G. Smith[1] & A.J. Christophers[2]

[1]*Statistical Centre, Peter MacCallum Cancer Institute, 481 Little Lonsdale Street, Melbourne, Victoria;* [2]*Department of Pharmacology, University of Melbourne, Victoria, Australia.*

**Summary** A case control study on patients with soft tissue sarcoma and malignant lymphoma was undertaken to test whether there was any association between these diseases and past exposure to chlorinated phenoxy acid herbicides or chlorophenols. It was carried out over the period 1982–1988 in Victoria, Australia. Thirty males with soft tissue sarcoma and 52 males with malignant lymphoma were matched by age, place of residence and sex with one population control and one cancer control each. Exposure was assessed by personal interviews conducted by an occupational hygienist. Exposures within 5 years prior to diagnosis of each matched case were ignored, both for the cases and their matched controls.

The estimated relative risks for definite or probable exposure to chlorinated phenoxy compounds or chlorophenols for at least 1 day were 1.0 (95% confidence interval (CI): 0.3–3.1) for soft tissue sarcoma and 1.5 (95% CI: 0.6–3.7) for malignant lymphoma. When the criterion for exposure was raised to more than 30 days, the estimated relative risks were 2.0 (95% CI: 0.5–8.0) for soft tissue sarcoma and 2.7 (95% CI: 0.7–9.6) for malignant lymphoma. Additional analyses were carried out for exposure of at least 1 day to phenoxy herbicides alone or chlorophenols alone. None of the estimated relative risks was significantly greater than unity.

A report of a case control study published in 1979 by Hardell and Sandström in Sweden was the first to claim an association between exposure to phenoxy herbicides and soft tissue sarcoma in humans (Hardell & Sandström, 1979). The authors also claimed to have found a link between exposure to chlorophenols and the same cancer and it was suggested that the carcinogenic agent in both types of chemicals could be a polychlorinated dibenzodioxin. This paper was soon followed by one reporting a similar study in a different area of Sweden (Eriksson *et al.*, 1981) and another one suggesting a link between exposure to these chemicals and malignant lymphoma (Hardell *et al.*, 1981). Prior to 1979 the claims of serious health effects from phenoxy herbicides had been confined to birth defects and other adverse pregnancy outcomes. The three Swedish studies were the first linking exposure of phenoxy herbicides with human cancer and they aroused considerable concern among public health authorities around the world.

In 1981 the Health Department of the State of Victoria, Australia, decided to support an investigation of the possible association between exposure to phenoxy herbicides or chlorophenols and the development of soft tissue sarcoma or malignant lymphoma, using a matched case-control study design. The design specified each case of soft tissue sarcoma or malignant lymphoma to be matched by age, sex and place of residence to one control without cancer drawn from the Victorian population and one control with cancer drawn from the same group of hospitals as the cases. Only living cases and controls were to be used and exposure was to be assessed by means of face-to-face interviews with the subjects themselves; no interviews with relatives were to be conducted. The results of this study are now reported.

The main chlorinated phenoxy herbicides used in Victoria have been 2,4-dichlorophenoxy acetic acid (2,4-D), 2,4,5-trichlorophenoxy acetic acid (2,4,5-T) and 4-chloro-2-methylphenoxy acetic acid (MCPA) and their esters and amines. Other chlorinated phenoxy agricultural chemicals used to a much lesser extent are 4-chlorophenoxy acetic acid (CPA), 2-[4-chloro-2-methylphenoxy] propionic acid (mecoprop), (±)-2-[2,4,5-trichlorophenoxy] propionic acid (fenoprop), sodium 2-[2,4-dichlorophenoxy] ethyl sulphate (2,4-DES-sodium), 4-[4-chloro-2-methylphenoxy] butyric acid (MCPB), 4-[2,4-dichlorophenoxy] butyric acid (2,4-DB) and methyl (±)-2-[4-(2,4-dichlorophenoxy) phenoxy] propionate (diclofop-methyl). At least two factories in Victoria have been involved in the manufacture of phenoxy herbicides and chlorophenols. Phenoxy herbicides have been widely used in Victoria to control broad-leaved weeds in cereal crops (wheat, oats, barley) and to control blackberry and other noxious weeds. Small amounts of chlorinated phenoxy compounds are used to prevent pre-harvest dropping of fruit.

Clofibrate, a chlorinated phenoxy drug, has been used to treat coronary heart disease, diabetes and high cholesterol.

The main chlorophenol used in Victoria is pentachlorophenol and its sodium salt. Other chlorophenols or salts used have been 2,4,6-trichlorophenate, sodium 2,3,4,6-tetrachlorophenate, 2-benzyl-4-chlorophenol, 3-methyl-4-chlorophenol, 3,5-dimethyl-4-chlorophenol, 3,5-dimethyl-2,4-dichlorophenol and bis(5-chloro-2-hydroxyphenyl) methane. Chlorophenols have been used mainly as wood preservatives, and also in leather tanning, as paint preservatives and anti mould treatments for walls, as disinfectants, as preservatives in adhesives, for slime control in paper pulp, for preserving size used on textiles, for mothproofing wool, as herbicides, and for various other fungicidal and bactericidal purposes.

### Subjects and methods

The population from which the cases and cancer controls were selected consisted of male cancer patients registered by the Victorian Cancer Registry after 1 January 1982 who were aged 30 years or more at registration, who were patients at any of six major Melbourne hospitals and who were still alive at the time of selection for the study. Since 1 January 1982 all cases of cancer diagnosed in Victoria, with the exception of non-melanoma skin cancer, have been required to be registered with the Cancer Registry. The study was restricted to six hospitals because the Cancer Registry was not permitted to contact patients before written informed consent of the patient had been obtained by the hospital concerned. The lower age limit of 30 years was specified in order to exclude young men whose cancers were unlikely to

Correspondence: J.G. Smith, Statistical Centre, Peter MacCallum Cancer Institute, 481 Little Lonsdale Street, Melbourne Victoria 3000, Australia.
Received 6 December 1990; and in revised form 5 August 1991.

be caused by occupational exposures, taking into account a reasonable latency period. Only living patients were included as it was considered that occupational data from relatives was likely to be incomplete and possibly inaccurate.

Cases were men with soft tissue sarcoma either coded as ICD 171 (World Health Organisation, 1977) or coded to other sites, and men with malignant lymphoma coded as ICD 200, ICD 201 or ICD 202. The study continued until interviews had been obtained from 30 patients with soft tissue sarcoma and 52 with malignant lymphoma. The histological diagnoses for the 82 cases are shown in Table I, together with their ICD codes. Histological types and ICD codes recorded on the Cancer Registry were confirmed from individual hospital records. No review of pathology specimens was undertaken. The cases were first diagnosed between 1976 and 1987 with 89% first diagnosed after 1 January 1982. The median age at diagnosis was 59 years (range 37 to 87) for soft tissue sarcoma cases, 39.5 years (range 28 to 57) for Hodgkin's disease cases and 59.5 years (range 30 to 87) for non-Hodgkin's lymphoma cases.

For each case, one control with another type of cancer was randomly selected by Cancer Registry staff, matching for sex, age within 3 years and Statistical Division of current residence. There are 12 Statistical Divisions in Victoria, with the city of Melbourne comprising one. Patients with leukaemia, multiple myeloma or sarcoma of bone were not eligible as cancer controls because the aetiology of these diseases may be similar to that of lymphoma or soft tissue sarcoma. The cancer controls had 23 different ICD codes from ICD 140 to ICD 194 with the largest groups being ICD 162 — bronchus and lung (17 controls), ICD 185 — prostate (nine controls), ICD 153 — colon (six controls) and ICD 154 — rectum (six controls).

Population controls were selected at random from the Electoral Register by Cancer Registry staff. It is compulsory for all Australian citizens over the age of 18 years to be on the Electoral Register. One population control was matched to each case by sex, age within 3 years and Statistical Division of current residence. Population controls who had had cancer (apart from non-melanoma skin cancer) were not eligible.

All selection and matching of cases and controls was done by Cancer Registry staff, independently of the principal investigators (the authors). Five controls differed by age from their matched cases by more than 3 years (3.1 to 5.4 years). One case was resident in New South Wales, although he attended a Melbourne hospital. He was matched with two controls resident in the nearest Statistical Division in Victoria. All other controls satisfied the matching criteria.

Written consent for a personal interview was sought from each case and cancer control. In the letters the purpose of the study was described as an investigation of possible associations between occupations and cancer. Herbicides or other chemicals were not mentioned. Initially, written consent was also required from population controls but the response rate was so low that the interviews with the eight population controls who consented were considered to be possibly biased and were discarded. From then on population controls were contacted in writing and given the opportunity to refuse to participate. If there was no written refusal, the interviewer then contacted the potential control by telephone or in person and, if verbal consent was obtained, the man was interviewed. This procedure meant that the interviewer could not be blind with regard to the population controls. However in a face-to-face interview involving a person's life history, blindness with respect to cancer status is virtually impossible.

Response rates in cases and controls were calculated after excluding 101 patients who were sent letters but who did not satisfy the eligibility criteria, 55 who were found to be dead by the time the letter would have been received, 14 who were reported to be no longer at the address on the Cancer Registry and 18 who were sent an incorrect letter which stated that either the patient or his relative could be interviewed (administrative error by one hospital). Two of those sent the incorrect letter were interviewed and included in the study as cases but were not included in the calculation of the response rates. Of the 301 remaining cancer patients who were sent letters, 187 agreed to be interviewed, 25 refused and 89 did not reply. Assuming that those who did not reply were in fact refusals, the response rates were 70% for cases and 56% for cancer controls. This was the most pessimistic view as some of those who did not reply might have never

**Table I** Histological types and ICD-9 codes of cases

| | *No. patients* | *ICD code* |
|---|---|---|
| *Histology of soft tissue sarcoma cases (30)* | | |
| Malignant fibrous histiocytoma | 9 | all 171 |
| Leiomyosarcoma | 5 | 152, 158, three 171 |
| Sarcoma, not otherwise specified (NOS) | 2 | both 171 |
| Liposarcoma, well differentiated | 2 | both 171 |
| Liposarcoma, NOS | 1 | 171 |
| Myxoid liposarcoma | 1 | 158 |
| Spindle cell sarcoma | 1 | 171 |
| Fibrosarcoma, NOS | 1 | 158 |
| Dermatofibrosarcoma, NOS | 1 | 173 |
| Epitheloid leiomyosarcoma | 1 | 171 |
| Synovial sarcoma, NOS | 1 | 171 |
| Clear cell sarcoma of tendons and aponeuroses | 1 | 171 |
| Kaposi's sarcoma | 1 | 173 |
| Malignant haemangiopericytoma | 1 | 171 |
| Chondrosarcoma NOS (extraosseous) | 1 | 171 |
| Malignant neurilemmoma | 1 | 171 |
| *Histology of malignant lymphoma cases (52)* | | |
| Hodgkin's disease - nodular sclerosis NOS | 6 | all 201 |
| Hodgkin's disease - mixed cellularity | 3 | all 201 |
| Hodgkin's disease - lymphocytic predominance | 1 | 201 |
| Reticulosarcoma | 15 | all 200 |
| Lymphocytic, poorly differentiated NOS | 3 | all 200 |
| Immunoblastic type | 2 | both 200 |
| Mixed lymphocytic-histiocytic NOS | 2 | both 200 |
| Lymphocytic, poorly differentiated, nodular | 8 | all 202 |
| Mixed lymphocytic-histiocytic, nodular | 4 | all 202 |
| Lymphoma, NOS | 2 | both 202 |
| Undifferentiated cell type NOS | 2 | both 202 |
| Nodular NOS | 2 | both 202 |
| Convoluted cell type NOS | 1 | 202 |
| Hairy cell leukaemia | 1 | 202 |

received their letters due to death or change of address. It is likely that some of the cases and cancer controls refused, or did not reply to, the request for interview because they were too ill. Eleven were later found to be within 3 months of death and another six gave other health-related reasons for refusing. Sixteen of those who agreed to interview could not be interviewed because of illness or administrative problems.

Of 160 population controls selected, 30 were not able to be contacted and six were ineligible because they had had cancer prior to the introduction of compulsory cancer registration. Of the remaining 124 contacted, 37 refused and 87 were interviewed, giving a response rate of 70%.

Nine eligible cancer controls and five eligible population controls who were interviewed were later excluded by Cancer Registry staff (without knowledge of the interview results). They were found not to match any cases or were superfluous to the requirement of one population control and one cancer control for each case (the control with the closest match was retained if two were available).

Of those who consented, 30 men with soft tissue sarcoma and 52 men with malignant lymphoma, as well as 164 matched controls (82 cancer controls and 82 population controls), were interviewed and included in the study. Unfortunately the distribution of interviews throughout the study was not uniform because of early administrative problems and the change to the method of obtaining population controls when it was realised that written consent was not feasible for these controls. Cases were interviewed between 1982 and 1988 with 80% interviewed prior to 1986, cancer controls between 1982 and 1988 with 18% interviewed prior to 1986 and population controls were all interviewed between 1986 and 1988. However all interviews were conducted by the same person (J.G.S.) who was blind as to the case control status of the cases and the cancer controls and who was unaware of the distribution of cases and controls at the time. The extra controls and ineligible subjects who were interviewed, but later excluded helped to ensure the interviewer's blindness. The percentage of subjects reporting exposure did not vary significantly with the year of interview over the 6 years of the study ($P = 0.63$, chi square test, 5 degrees of freedom) and there was no apparent trend over the years ($P = 0.69$, chi square test for trend). The lengthy duration of the study was due to the rarity of soft tissue sarcoma cases and some administrative problems.

The interviewer was an occupational hygienist with experience in pesticide exposures. In the interview, which usually lasted about 45 min, a comprehensive occupational history was obtained, plus details on education, leisure activities, and alcohol and tobacco consumption. Details on the nature and duration of exposure were sought if the subject reported any occupation or activity likely to involve the chemicals of interest. The fact that clofibrate could be a source of exposure to chlorinated phenoxy compounds was not realised until after the first ten subjects had been interviewed. From then on all subjects (except for one accidental omission) were asked about medications for diabetes, high blood pressure or high cholesterol to determine exposure to clofibrate. In addition to the occupational history, four specific questions were asked concerning work in the country or living on a farm, work with asbestos, use of pesticides, herbicides or wood preservatives, and work with lead. Positive answers were probed for further details. The questions on asbestos and lead were for camouflage purposes only. The subject was not told of the main purpose of the study, namely exposure to phenoxy herbicides or chlorophenols.

Exposures to phenoxy herbicides or chlorophenols or clofibrate were coded as none, possible or definite probable (Table II). This was done by the interviewer while still blind as to each subject's status and matched triad. In some instances this required extensive consultation with industry experts because subjects quoted superseded trade names or had worked in factories which were no longer in operation. For the analyses, exposures within 5 years prior to the year of diagnosis of a case were ignored, both for the case and his matched controls. A subject whose total lifetime exposure was less than 8 h (i.e. 1 day) was counted as not exposed.

The main analyses were carried out by comparing matched triads for exposures, i.e. case *vs* both matched controls. Soft tissue sarcomas and lymphomas were analysed separately. The method of conditional logistic regression described by Breslow and Day (1980, Chapter 7) was used to estimate relative risks and their approximate confidence intervals and test the null hypotheses. In the regression model for exposure to chlorinated phenoxy compounds or chlorophenols there were two indicator variables, one for possible exposure and one for definite exposure. The estimated relative risks reported are for definite exposure, adjusted for the estimated risk of possible exposure, i.e. with the possible exposure

**Table II** Exposures to chlorinated phenoxy compounds or chlorophenols (Numbers exposed for more than 30 days shown in parentheses)

| | *Soft tissue sarcoma* | | | *Malignant lymphoma* | | |
|---|---|---|---|---|---|---|
| *Exposure* | *Case* | *Pop. control* | *Cancer control* | *Case* | *Pop. control* | *Cancer control* |
| *Definite probable* | | | | | | |
| Spraying phenoxy herbicides | 5[a](4) | 4[b](1) | 2(1) | 7[d](3) | 9[b,d](1) | 4[c](2) |
| Other phenoxy acids | | 2(0) | | | | |
| PCP wood preservatives[e] | | | 1(1) | 1(0) | 1(0) | 1(0) |
| Sodium PCP as house painter | | | | 2(2) | | 1(1) |
| PCP in carpet glues as carpet layer | | | | 1(1) | | |
| Chlorophenol disinfectant as cleaner | | | | | 1(1) | |
| Clofibrate medication | | 1(1) | | | | |
| *Total definite probable* | 5(4) | 7(2) | 3(2) | 11(6) | 11(2) | 6(3) |
| *Possible* | | | | | | |
| Unknown disinfectants | 2(2) | | | 1(1) | | 1(1) |
| Unknown wood preservatives or possibly handling treated wood | 1(0) | | 2(2) | | | |
| Possibly exposed to PCP laurate at woollen mill | | 1(1) | | | | |
| Unknown herbicides | | | 1(0) | 3(0) | 2(0) | |
| Unknown chemicals in tanneries | | | | | 3(3) | |
| *Total possible* | 3(2) | 1(1) | 3(2) | 4(1) | 5(3) | 1(1) |

[a]One subject also made phenoxy acid derivatives in a laboratory (≤ 30 days); [b]One subject also used pentachlorophenol wood preservatives (≤ 30 days); [c]One subject also exposed to sodium pentachlorophenate in manufacture of animal glue (> 30 days, already counted as exposed > 30 days to phenoxy herbicides); [d]One subject also possibly exposed to sodium pentachlorophenate in latex glues as shoemaker (possible exposure > 30 days, definite exposure to phenoxy herbicides ≤ 30 days); [e]PCP = pentachlorophenol or pentachlorophenate.

variable in the model. The score statistic was used to test each null hypothesis. Because there were three levels of exposure to the chemicals of interest — none. possible. definite—the test statistic was compared with the chi square distribution with two degrees of freedom.

The *a priori* hypotheses of the study were whether exposure of at least 1 day to chlorinated phenoxy herbicides or chlorophenols was associated with the development of soft tissue sarcoma or malignant lymphoma. Subsequent analyses were also carried out to investigate whether there was any risk associated with exposure of at least 1 day to chlorinated phenoxy herbicides alone or chlorophenols alone and whether there was any risk associated with exposure to either group of chemicals for more than 30 days.

In addition. analyses of the matched pairs. cases *vs* population controls and cases *vs* cancer controls. were carried out. For the matched pairs analyses. the classical methods described by Breslow and Day (1980. p. 182) were used to estimate relative risks and test the null hypotheses. The score test statistic was calculated and compared to the chi square distribution with two degrees of freedom. Approximate confidence intervals were calculated using conditional logistic regression (program STRAT. Breslow & Day. 1980 or software package GLIM. Adena & Wilson. 1982). The confidence intervals reported for the matched pairs are of limited validity. because no adjustment has been made for the fact that the two comparisons made to test each hypothesis are not independent. i.e. the same cases are used in each matched pair comparison.

Matched pair analyses of cases *vs* population controls only were carried out to test for possible effects of smoking tobacco (non-smoker. past smoker or current smoker of at least one cigarette per day for as long as 6 months) and drinking alcohol (non-drinker. past drinker or current drinker of more than 100 grams of alcohol per year). A 5 year latency period prior to the year of diagnosis of the matched cases was applied as in previous analyses. Comparisons of cases with cancer controls were not made for smoking and drinking because several of the cancers among the cancer controls are known to be strongly associated with tobacco and alcohol. namely lung. larynx. kidney and bladder. In each of the two regression models for smoking and drinking there were two indicator variables. one for past smoking drinking and one for current smoking drinking. For these analyses the estimated relative risk for current smokers after adjustment for the risk for past smokers is reported and the estimated relative risk for past smokers after adjustment for the risk for current smokers is reported. Similarly the estimated relative risks are reported for current and past drinkers.

As a hypothesis generating exercise. all occupations of at least 5 years' duration prior to the date of interview were coded according to the Australian Standard Classification of Occupations (Castles. 1986). Cases and controls were compared to see if there were any significant clusters of soft tissue sarcoma or malignant lymphoma cases in each occupation. A latency period of 5 years prior to the year of diagnosis of the matched cases was applied. Any occupation which had a cluster of five or more cases was then analysed using the classical method for matched triads and dichotomous exposures described by Breslow & Day (1980. p. 169).

### Results

Forty-three men (16 cases. 18 population controls. nine cancer controls) were definitely or probably exposed and 17 men (seven cases. six population controls. four cancer controls) were possibly exposed to chlorinated phenoxy compounds or chlorophenols for at least 1 day prior to 5 years before the year of diagnosis of the case in each matched triad. The exposures are listed in Table II. The total amount of exposure for those involved in spraying phenoxy herbicides ranged from 8 h to 122 weeks. the latter being the length of exposure of a population control. The total amount of exposure for those using pentachlorophenol as a wood preservative ranged from 14 h to 190 days. the latter being the length of exposure of a cancer control. There were no significant differences between population controls and cancer controls with respect to definite exposure (soft tissue sarcoma controls 7 30 *vs* 3 30. $P = 0.30$; malignant lymphoma controls 11 52 *vs* 6 52. $P = 0.29$. Fisher exact test. two tailed). Hence the cancer controls and population controls were combined and matched triad analyses were done.

The data were tabulated according to the exposure of each subject in the triad. The matched triad data for testing the two main hypotheses of the study are shown in Table III. the estimated relative risks with approximate 95% confidence intervals are shown in the first line of Table IV. Neither of the relative risks is significantly different from unity ($P > 0.6$ for both).

Analyses were also carried out for phenoxy herbicides and chlorophenols separately. using the same exposure criteria. In the phenoxy herbicide analysis. exposure to chlorophenols or clofibrate was counted as no exposure. and in the chlorophenol analysis. exposure to phenoxy herbicides or clofibrate was counted as no exposure. The estimated relative risks are shown in the second and third lines of Table IV. None is statistically significant ($P > 0.4$ for all comparisons).

An analysis was also carried out for exposure of more than 30 days to either chlorinated phenoxy compounds or chlorophenols prior to 5 years before the year of diagnosis of the matched cases (a subject exposed for 30 days or less was counted as not exposed). The estimated relative risks are shown in the fourth line of Table IV. They are higher than the risks for one day's minimum exposure but they are still not statistically significant ($P > 0.2$ for both comparisons). Because of the small numbers exposed for more than 30 days. the confidence intervals are relatively wide.

The results of the matched pair analyses are shown in Table V. The confidence intervals are wider than those obtained for the matched triad analyses because only half the number of controls are used in each comparison and because pairs in which both the case and his matched control are

**Table III** Exposure of matched triads to chlorinated phenoxy compounds or chlorophenols for at least one day. 5 years' latency period

| | Exposure of controls | | | | | | |
|---|---|---|---|---|---|---|---|
| *Exposure of case* | *Both none* | *1 none. 1 possible* | *Both possible* | *1 none. 1 definite* | *1 possible. 1 definite* | *Both definite* | *Total* |
| *A. Soft tissue sarcoma* | | | | | | | |
| None | 10 | 4 | 0 | 8 | 0 | 0 | 22 |
| Possible | 2 | 0 | 0 | 1 | 0 | 0 | 3 |
| Definite | 4 | 0 | 0 | 1 | 0 | 0 | 5 |
| Total | 16 | 4 | 0 | 10 | 0 | 0 | 30 |
| *B. Malignant lymphoma* | | | | | | | |
| None | 25 | 3 | 0 | 7 | 0 | 2 | 37 |
| Possible | 2 | 0 | 0 | 1 | 0 | 1 | 4 |
| Definite | 6 | 2 | 0 | 2 | 1 | 0 | 11 |
| Total | 33 | 5 | 0 | 10 | 1 | 3 | 52 |

equally exposed are ignored in the analysis. (There were no triads in which the case and both his controls were equally exposed so the matched triad analysis was able to make full use of the data.) None of the estimated relative risks for the matched pairs was significantly different from unity ($P > 0.1$ for all comparisons).

The estimated relative risks for smoking tobacco or drinking alcohol are shown in Table VI. No statistically significant associations were found ($P > 0.1$ for all comparisons).

When all occupations were examined for significant clusters of cases it was found that the 30 soft tissue sarcoma cases had 67 occupations of at least 5 years' duration prior to the date of interview, among which there were 52 different occupation codes. The 52 malignant lymphoma cases had 111 occupations of at least 5 years' duration with 80 different occupation codes. After applying a 5 years' latency period and analysing the data as matched triads, no statistically significant clusters were found for any occupation when coded to a six digit level of specialization ($P > 0.1$). When similar occupations were combined to the extent that the first four digits of the occupation codes were the same, there was still no significant clusters ($P > 0.1$). However the study was designed to test two specific hypotheses rather than generate new ones, and the number of cases was too small to be likely to find statistically significant clusters unless a very strong association were present.

Thirteen cases (16%) and 25 cancer controls (30%) volunteered opinions as to the causes of their cancers although this was not asked by the interviewer. No cases, who gave an opinion, suspected phenoxy herbicides or chlorophenols and only one cancer control did so. Thus recall bias on the part of the subjects was not apparent.

### Discussion

The results of this study do not support the hypotheses that exposure to chlorinated phenoxy herbicides or chlorophenols causes soft tissue sarcoma or malignant lymphoma. In the main analysis the relative risks of developing soft tissue sarcoma or malignant lymphoma following exposure of at least 1 day to these chemicals were estimated to be 1.0 (95% CI: 0.3–3.1) and 1.5 (95% CI: 0.6–3.7) respectively.

When the relative risks were estimated from the matched pairs, the risks calculated from cases *vs* cancer controls were generally higher than the risks estimated from cases *vs* population controls, although none of the relative risks was statistically significant at the 0.1 level. If a higher relative risk had been found when comparing cases with population controls than when comparing cases with cancer controls, it could have been explained in terms of recall bias. As it is, however, there is no obvious explanation for the variation between estimates of relative risk apart from chance.

The number of subjects exposed was too small to be able to estimate with reasonable power the relative risks for exposure of more than 30 days to phenoxy herbicides or chlorophenols or for exposure of at least 1 day to chlorophenols alone.

In the three early Swedish studies the estimated (matched) relative risks for exposure of at least 1 day to chlorinated phenoxy herbicides or chlorophenols were 6.2 and 5.1 for soft tissue sarcoma (Hardell & Sandström, 1979; Eriksson *et al.*, 1981) and 6.0 for malignant lymphoma (Hardell *et al.*, 1981). In recent years the Swedish researchers have carried out two more case-control studies on soft tissue sarcoma in which the estimated relative risks were much lower. In a study reported in 1988 the estimated relative risk (unmatched, stratified) for phenoxy acetic acids alone was 3.3 and for chlorophenols alone was less than unity (actual estimate not reported: Hardell & Eriksson, 1988). Conversely, in a study published in 1990, no significant risk was found for phenoxy herbicides alone (RR 1.3, 95% CI: 0.7–2.6) but the estimated relative risk for chlorophenols alone was significant (RR 5.2, 95% CI: 1.7–16.3; Eriksson *et al.*, 1990). The estimated relative risk (matched) for exposure to either phenoxy herbicides or chlorophenols was 1.8 (95% CI: 1.1–3.0) in the 1990 study.

Many other cohort and case-control studies have been carried out to investigate the possible association between phenoxy herbicides or chlorophenols and soft tissue sarcoma or lymphoma. Of at least 18 studies of cohorts or workers known to be exposed to phenoxy herbicides or chlorophenols, only one has found a statistically significant association with either of these cancers. This is a study by Lynge (1985) who found a significant excess of soft tissue sarcomas in workers employed in manufacturing phenoxy herbicides in Denmark. A recent nationwide cohort study of 12 USA plants manufacturing chemicals contaminated with 2,3,7,8-tetrachlorodibenzo-*p*-dioxin found an increased incidence of soft tissue sarcoma but the statistical significance of the results are in doubt because of misclassification of soft tissue sarcoma on death certificates (Fingerhut *et al.*, 1991).

Case control studies investigating actual exposures to the chemicals of interest, rather than presumptive exposures associated with occupations or military service in Vietnam, have been carried out in New Zealand (Smith *et al.*, 1983,

**Table IV** Estimated relative risks for exposure to chlorinated phenoxy compounds or chlorophenols, 5 years' latency period, matched triads, (95% confidence intervals in parentheses)

| *Exposure* | *Soft tissue sarcoma* | *Malignant lymphoma* |
|---|---|---|
| Phenoxy herbicides or chlorophenols ⩾ 1 day | 1.0 (0.3–3.1) | 1.5 (0.6–3.7) |
| Phenoxy herbicides ⩾ 1 day | 1.3 (0.4–4.1) | 1.1 (0.4–3.0) |
| Chlorophenols ⩾ 1 day | 0 (–)[a] | 1.4 (0.3–6.1) |
| Phenoxy herbicides or chlorophenols > 30 days | 2.0 (0.5–8.0) | 2.7 (0.7–9.6) |

[a]No soft tissue sarcoma cases were exposed to chlorophenols so confidence interval could not be calculated.

**Table V** Estimated relative risks for exposure to chlorinated phenoxy compounds or chlorophenols, 5 years' latency period, matched pairs, (95% confidence intervals in parentheses)[a]

| *Exposure* | *STS* vs *pop. cont.* | *STS* vs *cancer cont.* | *ML* vs *pop. cont* | *ML* vs *cancer cont.* |
|---|---|---|---|---|
| PH or CP ⩾ 1 day | 0.7 (0.2– 2.6) | 1.7 (0.4– 7.0) | 0.9 (0.4– 2.4) | 2.6 (0.8– 8.8) |
| PH ⩾ 1 day | 0.8 (0.2– 3.7) | 2.5 (0.5–12.9) | 0.8 (0.3– 2.2) | 1.8 (0.5– 6.0) |
| CP ⩾ 1 day | 0 – | 0 – | 1.2 (0.3– 5.4) | 3.0 (0.2–44.9) |
| PH or CP > 30 days | 2.0 (0.4–10.9) | 2.0 (0.4–10.9) | 3.0 (0.6–14.9) | 3.0 (0.5–17.0) |

[a]Abbreviations: STS = soft tissue sarcoma, ML = malignant lymphoma, pop. = population, cont. = controls, PH = phenoxy herbicides, CP = chlorophenols.

**Table VI** Estimated relative risks for smoking tobacco or drinking alcohol, 5 ve[illegible]s' latency, cases *vs* population controls, (95% confidence intervals in parentheses)[a]

| | *Soft tissue sarcoma* vs *population controls* | *Malignant lymphoma* vs *population controls* |
|---|---|---|
| Current smoker | 2.8 (0.9– 9.0) | 2.2 (0.7 –6.7) |
| Past smoker | 1.1 (0.3– 3.8) | 2.2 (0.7 –7.1) |
| Current drinker | 2.3 (0.6– 8.9) | 0.6 (0.2 –2.0) |
| Past drinker | 2.9 (0.4–25.0) | 0.4 (0.04–3.3) |

[a]Smoker = smoking as much as one cigarette per day for as long as 6 months, drinker = consuming more than 100 grams of alcohol per year.

1984; Smith & Pearce, 1986; Pearce *et al.*, 1986, 1987), Kansas, USA (Hoar *et al.*, 1986), Western Washington State, USA (Woods *et al.*, 1987) and Northern Italy (Vineis *et al.*, 1986). None of the main results of these studies showed a statistically significant association except for the study by Hoar *et al.* (1986) which reported a significant association between non-Hodgkin's lymphoma and phenoxy herbicides (odds ratio 2.2, 95% CI: 1.2–4.1). Hoar *et al.* found no increased risk of soft tissue sarcoma or Hodgkin's disease. The largest case control study, by Woods *et al.* (1987), reported an odds ratio of 0.9 (95% CI: 0.5–1.5) for non-Hodgkin's lymphoma and phenoxy herbicides and no increased risk of soft tissue sarcoma (actual odds ratio not reported).

In the present study considerable efforts were made to obtain the most accurate exposure data possible by the use of face-to-face interviews and by obtaining data from the subjects themselves, rather than next-of-kin of deceased subjects. The matching of cases and controls was maintained throughout the analysis to maximise the power to detect increased relative risks if they existed. The opinions expressed by subjects as to the causes of their cancer indicated a lack of any recall bias.

Two weaknesses in the study are its small size and the relatively low response rates. Larger sample sizes would have made the study impractical. The rarity of soft tissue sarcoma meant that it took over 5 years to accrue 30 living soft tissue sarcoma patients who would consent to be interviewed. It was considered undesirable to obtain larger numbers of cases by interviewing relatives of dead patients as the data would be considerably less reliable. The sample sizes were sufficient to detect a relative risk of 5 at the 0.05 level of significance with power of 90% for soft tissue sarcoma and 99% for malignant lymphoma.

The response rates were low compared to the Swedish studies by Hardell and his colleagues but not very much lower than in the New Zealand case-control studies (79% to 88%) (Smith *et al.*, 1983, 1984; Pearce *et al.*, 1986, 1987). It is understandable that response rates will decrease with the amount of effort required. Face-to-face interviews are more demanding than postal questionnaires which were used in Sweden or telephone interviews which were used in New Zealand. Age group ($<65$ *vs* $\geqslant 65$ years) and place of residence (city *vs* country) were not significantly associated with response rate. Response rates for cancer patients varied with different hospitals and gradually decreased over the period of the study, probably because of waning enthusiasm by hospital staff involved in sending out letters and following up non-responders. This is unlikely to have introduced a bias with respect to the exposures of interest however.

It proved to be too difficult to arrange for pathological review of soft tissue sarcoma specimens. In a recent study a pathological review of soft tissue sarcoma diagnoses (Alvegård & Berg, 1989), 5% of the soft tissue sarcomas reviewed were re-diagnosed as non-sarcomatous tumours. This paper referred to two other studies in which 7% of 'soft tissue sarcomas' and 6% of 'bone and soft tissue sarcomas' were non-sarcomatous tumours respectively. It therefore seems possible that, in our 30 cases diagnosed as soft tissue sarcoma, one or two cases may have been wrongly diagnosed. It is unlikely that this would make a significant difference to the main conclusion.

One possible source of bias in the comparison between cases and population controls was the fact that the cases were drawn from a population of public (non fee paying) patients at six Melbourne hospitals, whereas the population controls were drawn from all registered voters in the State of Victoria (all social classes). Most patients with cancer in Victoria would attend a Melbourne hospital at some stage in their disease. Matching for statistical division of residence helped to eliminate a possible selection bias introduced by the hospital locations and catchment areas. However there remained a potential selection bias due to possible social class differences between public (non-fee paying) patients and the population controls.

One measure of social class in Australia is educational level. Cases had slightly more education than their matched cancer controls, although the difference was not statistically significant ($P = 0.08$ for age of leaving school, $P = 0.4$ for highest qualification ever obtained, Wilcoxon signed rank test). Cases had significantly less education than their matched population controls ($P = 0.006$ for age of leaving school, $P = 0.02$ for highest qualification ever obtained). However there was no association found between definite exposure to chlorinated phenoxy compounds or chlorophenols (categorised as none, 1–30 days, $>30$ days) and age of leaving school (dichotomised as $\leqslant 15$ years and $>15$ years) ($P = 0.92$, chi square test for trend) or between definite exposure and highest qualification ever obtained (dichomotised as primary or secondary school only *vs* trade certificate, diploma or degree) ($P = 0.62$, chi square test for trend). If it can be assumed that educational level is a good measure of social class, then it can be concluded that the social class differences between cases and controls did not affect the relative risks concerning exposure to chlorinated phenoxy compounds or chlorophenols.

This study has found no statistically significant association between exposure to phenoxy herbicides or chlorophenols and the development of soft tissue sarcoma and lymphoma. However it is a relatively small study and the findings should be viewed as one piece of evidence in the large and growing literature concerning this question.

**Note:** Details of this study are included in a thesis by one of us (J.G.S.) submitted to the University of Sydney.

We are most grateful to the staff of the Victorian Cancer Registry who carried out the selection and matching of cases and controls and correspondence with hospitals, in particular Ms Helen Handsjuk, Dr Graham Giles, Ms Vicki Higgins, Ms Roseanne Evans and Ms Alison Dodds. The cooperation of the Directors of Medical Services, medical records staff and other staff at the following hospitals is much appreciated: Peter MacCallum Cancer Institute, Alfred Hospital, Prince Henry's Hospital, St Vincent's Hospital, Austin Hospital and Royal Melbourne Hospital. The assistance of Dr J.D. Mathews in the preparation of the protocol, and the cooperation of cancer patients and healthy men, who agreed to be interviewed, are gratefully acknowledged. The ancillary expenses of the study were supported by a grant from the Health Department of Victoria.

### References

ADENA, M.A. & WILSON, S.R. (1982). *Generalised Linear Models in Epidemiological Research. Case-control Studies.* The Instat Foundation for Statistical Data Analysis: Sydney.

ALVEGÅRD, T.A. & BERG, N.O. (1989). Histopathology peer review of high-grade soft tissue sarcoma: the Scandinavian Sarcoma Group experience. *J. Clin. Oncol.*, **7**, 1845.

BRESLOW, N.E. & DAY, N.E. (1980). *Statistical Methods in Cancer Research. Volume 1-The Analysis of Case-control Studies*. International Agency for Research on Cancer: Lyon.

CASTLES, I. (1986). *Australian Standard Classification of Occupations. First edition*. Australian Bureau of Statistics: Canberra.

ERIKSSON, M.E., HARDELL, L., BERG, N.O., MÖLLER, T. & AXELSON, O. (1981). Soft-tissue sarcomas and exposure to chemical substances: a case-referent study. *Br. J. Ind. Med.*, **38**, 27.

ERIKSSON, M., HARDELL, L. & ADAMI, H.-O. (1990). Exposure to dioxins as a risk factor for soft tissue sarcoma: a population-based case-control study. *J. Natl Cancer Inst.*, **82**, 486.

FINGERHUT, M.A., HALPERIN, W.E., MARLOW, D.A. & 7 others (1991). Cancer mortality in workers exposed to 2,3,7,8-tetrachlorodibenzo-*p*-dioxin. *New Engl. J. Med.*, **324**, 212.

HARDELL, L. & SANDSTRÖM, A. (1979). Case-control study: soft-tissue sarcomas and exposure to phenoxyacetic acids or chlorophenols. *Br. J. Cancer*, **39**, 711.

HARDELL, L., ERIKSSON, M., LENNER, P. & LUNDGREN, E. (1981). Malignant lymphoma and exposure to chemicals, especially organic solvents, chlorophenols and phenoxy acids: a case-control study. *Br. J. Cancer*, **43**, 169.

HARDELL, L. & ERIKSSON, M. (1988). The association between soft tissue sarcomas and exposure to phenoxyacetic acids. *Cancer*, **62**, 652.

HOAR, S.K., BLAIR, A., HOLMES, F.F. & 4 others (1986). Agricultural herbicide use and risk of lymphoma and soft-tissue sarcoma. *JAMA*, **256**, 1141.

LYNGE, E. (1985). A follow-up study of cancer incidence among workers in manufacture of phenoxy herbicides in Denmark. *Br. J. Cancer*, **52**, 259.

PEARCE, N.E., SMITH, A.H., HOWARD, J.K., SHEPPARD, R.A., GILES, H.J. & TEAGUE, C.A. (1986). Non-Hodgkin's lymphoma and exposure to phenoxyherbicides, chlorophenols, fencing work, and meat works employment: a case-control study. *Br. J. Ind. Med.*, **43**, 75.

PEARCE, N.E., SHEPPARD, R.A., SMITH, A.H. & TEAGUE, C.A. (1987). Non-Hodgkin's lymphoma and farming: an expanded case-control study. *Int. J. Cancer*, **39**, 155.

SMITH, A.H., FISHER, D.O., GILES, H.J. & PEARCE, N. (1983). The New Zealand soft tissue sarcoma case-control study: interview findings concerning phenoxyacetic acid exposure. *Chemosphere*, **12**, 565.

SMITH, A.H., PEARCE, N.E., FISHER, D.O., GILES, H.J., TEAGUE, C.A. & HOWARD, J.K. (1984). Soft tissue sarcoma and exposure to phenoxyherbicides and chlorophenols in New Zealand. *J. Natl Cancer Inst.*, **73**, 1111.

SMITH, A.H. & PEARCE, N.E. (1986). Update on soft tissue sarcoma and phenoxyherbicides in New Zealand. *Chemosphere*, **15**, 1795.

VINEIS, P., TERRACINI, B., CICCONE, G. & 8 others (1986). Phenoxy herbicides and soft-tissue sarcomas in female rice weeders. A population-based case-referent study. *Scand. J. Work Environ. Health*, **13**, 9.

WOODS, J.S., POLISSAR, L., SEVERSON, R.K., HEUSER, L.S. & KULANDER, B.G. (1987) Soft tissue sarcoma and non-Hodgkin's lymphoma in relation to phenoxyherbicide and chlorinated phenol exposure in western Washington. *J. Natl Cancer Inst.*, **78**, 899.

WORLD HEALTH ORGANISATION (1977). *International Classification of Diseases, 1975 Revision, Volume 1 (9th ed)*. World Health Organisation: Geneva.